# Proceedings of the 3rd annual Acute Cardiac Unloading and REcovery (A-CURE) symposium

**DOI:** 10.1186/s12872-019-1000-z

**Published:** 2019-02-07

**Authors:** Uma Chandrasekaran, Daniel Burkhoff, Kiyotake Ishikawa, Lija Swain, Kenji Sunagawa, Jacob Møller, Carlos Santos-Gallego, Shiva Annamalai, James Udelson, Ralf Westenfeld, Navin Kapur, Xiaoying Qiao, Julian Wiora, Andreas Schäfer, Alexander Bernhardt, Ajar Kochar, Robert Kloner, Haroon Faraz

**Affiliations:** 1 Abiomed, Inc., Danvers, MA USA; 2 Cardiovascular Research Foundation and Columbia University, New York City, NY USA; 3 Icahn School of Medicine at Mount Sinai, New York City, NY USA; 4 Tufts Medical Center, Boston, MA USA; 5 Kyushu University, Fukuoka, Japan; 6 Odense University Hospital, Odense, Denmark; 7 University Hospital Düsseldorf, Düsseldorf, Germany; 8 Hannover Medical School, Hannover, Germany; 9 University Heart Center Hamburg, Hamburg, Germany; 10 Duke Clinical Research Institute, Durham, NC USA; 11 Huntington Medical Research Institute & University of South California, Pasadena, CA USA; 12 Hackensack University Medical Center, Hackensack, NJ USA

## Foreword

Welcome to this special supplement devoted to the proceedings of the 3rd Annual Acute Cardiac Unloading and REcovery (A-CURE) Working Group meeting, which was held on November 9, 2018, in Chicago, USA. The A-CURE Working Group is comprised of leading academic experts in clinical and basic cardiac research who are dedicated to advancing the science and clinical application of acute cardiac unloading. This meeting brought together experts from multiple disciplines, including interventional cardiologists, heart failure specialists, cardiac surgeons, molecular biologists, and biomedical engineers. The 2018 Symposium featured talks and posters that highlighted cutting-edge advances in the field of acute cardiac unloading that have taken place since the conclusion of the 2017 A-CURE Symposium in Barcelona, Spain.

Cardiac disease states such as myocardial infarction (MI), myocarditis, cardiomyopathy, and cardiogenic shock impair the ability of the heart to pump blood, resulting in end organ failure and, ultimately, death. Pharmacological therapies for these disease states aim to maintain cardiac output but, in the process, impose further stress on the heart. Additional treatment strategies are needed. The A-CURE Symposium focused on the basic science and clinical application of new technologies. Acute cardiac unloading decreases myocardial oxygen consumption and maximizes the ability of the heart to rest and recover after damage. Mechanical unloading employs percutaneous ventricular assist devices such as the FDA-approved Impella family of devices, to decrease the physical workload of the heart.

This supplement features a number of presentations covering a broad range of subjects related to cardiac unloading. The first session of the symposium was devoted to the advances in basic and preclinical science of acute unloading and myocardial salvage. Topics discussed during the presentations ranged from influence of acute unloading on intercellular and inter-organ communication through exosome-based signaling to preservation of mitochondrial structure and function post-myocardial infarction (MI). New models of cardiogenic shock and investigations demonstrating enhanced collateral blood flow with acute unloading to reduce infarct size were also discussed.

In the keynote lecture, James Udelson focused on the physiologic and pathologic basis of left ventricular remodeling and the lessons learned from clinical trials in the field of chronic heart failure.

The second session of the symposium focused on clinical research programs of cardiac unloading. Wide spectrum of clinical studies presented included cardio-renal system interaction with effect of hemodynamic support on acute kidney injury, outcomes associated with adoption of standardized protocol for treatment in cardiogenic shock, and the first-in-man experience with the new Impella 5.5 heart pump.

The afternoon’s presentations had a stronger focus on the clinical translation of left ventricular (LV) unloading. The temporal trends and patterns of the incidence of new heart failure (HF) post-MI and the potential of cardiac cell transplantation to fashion an external auxiliary circulatory pump were presented. The meeting concluded with two talks focused on the first exploratory study testing the safety and feasibility of LV unloading and delayed reperfusion, the door to unload (DTU) in ST-elevation in myocardial infarction (STEMI) pilot trial. The rationale, challenges, and learnings from the DTU in STEMI pilot trial were discussed.

The presentations highlighted the exciting new developments and represent substantial advances in the field of acute myocardial unloading and recovery that have developed in the last year. The A-CURE Working Group meeting is unique in involving a diverse group of experts from multiple disciplines within an open, constructive, and intimate public setting.

We hope that you find this supplement informative and interesting.

## The state of the field: our current understanding of ventricular unloading

### Presented by Daniel Burkhoff, MD, PhD

Dr. Burkhoff commenced the meeting by stating the mission of the A-CURE symposium, which is to advance the science and mechanistic understanding of acute cardiac unloading and support the translation of basic and clinical research into therapies aimed at heart muscle recovery. He noted that since the inception of the A-CURE Working Group 4 years ago, the mission and application of the science has rapidly progressed into the clinical setting, particularly in reference to the recent completion of the Door To Unload in STEMI clinical trial, an idea initially proposed at the first annual A-CURE Symposium in 2016.

Dr. Burkhoff presented a brief history of the A-CURE symposium, which began with the first faculty meeting in Paris in 2015. This was followed by the 1st Annual A-CURE Symposium in Rome (2016) and the 2nd Annual Symposium in Barcelona (2017). He added that following this year’s meeting in Chicago (2018), the next meeting will be held in Paris in 2019. He highlighted the steady year-over-year growth in attendees of the Annual Symposia.

**Fig. 1:**
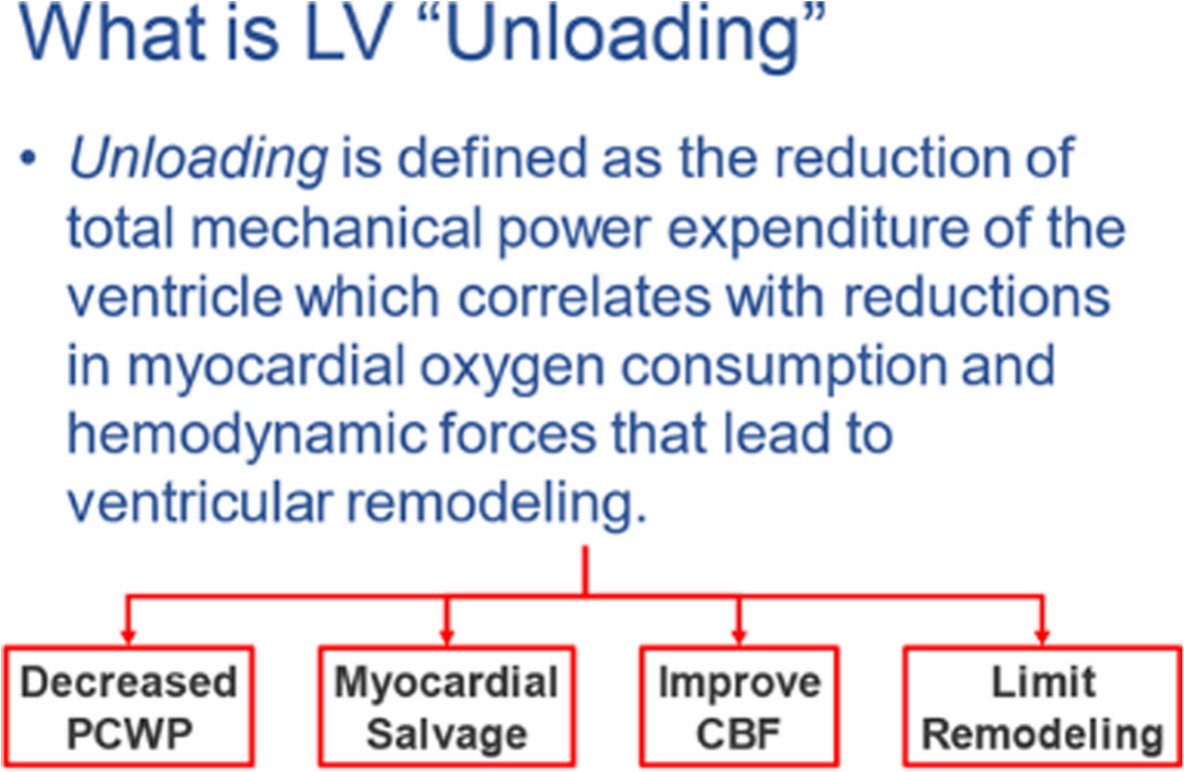
Definition of myocardial unloading and benefits during myocardial infarction. LV = left ventricular; PCWP = pulmonary capillary wedge pressure; CBF = coronary blood flow

He guided the audience through a brief discussion of the proposed formal definition of left ventricular (LV) unloading. He put forth that LV unloading is defined as the reduction of total mechanical power expenditure of the ventricle which correlates with a reduction in myocardial oxygen consumption and hemodynamic forces that lead to ventricular remodeling [1]. He emphasized that myocardial unloading is not only about mechanics but also about metabolism and oxygen consumption. So, the goal of ventricular unloading is twofold: first to achieve myocardial salvage and second to prevent cardiac remodeling and subsequent heart failure. When the goal of myocardial unloading is achieved, it results in decreased left ventricular pressure and volume with the subsequent reduction in pulmonary capillary wedge pressure (PCWP), salvage of myocardium, improved coronary blood flow (CBF), and it limits remodeling (Fig. 1).

While the concept of unloading has been discussed for decades, he highlighted that the scientific investigation and application of unloading emerged from the seminal work by E. Braunwald and M. Pfeffer in 1985. This team first demonstrated the benefits of LV unloading using captopril, an angiotensin-converting enzyme inhibitor, in a rat model of myocardial infarction (MI) [2]. This pre-clinical finding was tested in a clinical trial that showed a significant reduction in end-diastolic pressure and PCWP with a resultant decrease in ventricular dilation with captopril [3]. These studies were followed by additional trials such as SAVE that were critical in establishing pharmacological therapies for the treatment of MI.

However, there are inherent limitations to myocardial unloading using pharmacological therapies. Importantly, more unloading of LV pharmacologically to leads to more compromise in aortic pressure and cardiac output. Hence, the use of pharmacological therapies has been limited as a route to LV unloading. On the other hand, use of a percutaneous ventricular assist device (pVAD) like Impella can simultaneously unload the ventricle, reduce the workload of the heart, and increase the end-organ perfusion while maintaining cardiac output. As early as 1992, Smalling et al. showed improved regional myocardial blood flow, LV unloading, and infarct salvage using the Hemopump in a dog-model of MI [4]. Meyns et al. confirmed the above findings using Impella in a sheep model of ischemia-reperfusion [5]. They demonstrated that LV unloading using Impella during ischemia and reperfusion reduced myocardial oxygen consumption resulting in reduced infarct size.

Through the work of Dr. Suga and Dr. Sagawa, we have learned about the determinants of myocardial oxygen consumption (MVO_2_) in the context of pressure-volume loops. MVO_2_ is linearly related to pressure-volume area (PVA). PVA is the sum of the stroke work and the potential energy, i.e. the energy that is stored in the myocardial filaments after contraction rather than being released as external work. It is also important to note that even when the heart is producing no external work, it still consumes energy, largely due to calcium cycling, but also due to basal metabolism. As drugs increase contractility, they increase the mechanical work of the heart resulting in increased MVO_2_, which is detrimental in the setting of MI where energy supply is limited. In addition, heart rate is another determinant of MVO_2_. Recent work by Sunagawa et al. demonstrated that the combination of mechanical unloading and ivabradine (a bradycardic agent), synergistically reduces MVO_2_ and significantly reduces infarct size in a dog-model of ischemia-reperfusion [6].

Right now, studies are underway in humans to see if mechanical unloading with devices will reduce infarct size. Once that is well established, it will be interesting to see if the combination of drugs such as beta-blockers and ivabradine with mechanical circulatory support devices will further reduce infarct size and improve outcomes. Finally, Dr. Burkhoff highlighted that the studies from the Kapur lab are not only reproducing the findings of a reduction in infarct size with LV unloading but also starting to unravel the underlying molecular mechanism of cardioprotection by LV unloading [7].

Dr. Burkhoff concluded with a mention that this year’s meeting was a prelude to the results of the 1st randomized clinical study of unloading to reduce infarct size, the door to unloading (DTU) trial. He was delighted to be witnessing and playing a part in the dawn of a new era in clinical cardiology.

## Cardiac uptake and release of exosomes during altered left ventricular load in ischemic HF

### Presented by Kiyotake Ishikawa, MD

Dr. Ishikawa’s lab is interested in the studying the non-LV effects of LV unloading. Although most of the effects of LV unloading can be explained by a change in hemodynamics, a potential role of humoral effects has not been studied and may play a vital role in cross-organ communication during LV unloading. Dr. Ishikawa’s lab is investigating the exosomal microRNA-mediated regulation of local and remote communication [8] during LV unloading in a porcine model of percutaneously induced anterior myocardial infarction (MI). One week after MI, pigs underwent LV unloading using Impella CP for 2 h or LV over-loading following percutaneous induction of aortic regurgitation. MicroRNAs packaged in the exosomes extracted from blood samples collected from the coronary artery were compared with those from the coronary sinus, both before and after cardiac unloading/overloading.

A total of 127 microRNAs were identified among 3 pigs receiving LV unloading versus 220 microRNAs after LV overloading. Of the 127 microRNAs in the LV unloading group, 39 microRNAs were taken up by and 7 were released from the heart. Of the 220 microRNAs in the LV overloading group, 33 were taken up by and 32 were released from the heart. Six microRNAs showed an opposite transcardiac gradient after LV unloading versus LV overloading, that is increased uptake after overloading and increased release after unloading. In addition, they also performed a direct comparison of microRNAs in the coronary artery or coronary sinus blood before and after unloading or overloading and found significant difference in the microRNAs in the coronary sinus alone.

In conclusion, many exosomal microRNAs show transcardiac gradient and opposite changes in some of the microRNAs after unloading versus overloading suggests load-dependence, and may play a role in cross-organ communication. This is the first study investigating how acute ventricular unloading may regulate exosome-mediated cellular and organ communication. These preliminary results suggest that mechanical cardiac unloading may alter intra- and inter-organ communication. The direct consequences of this remains to be investigated.

## Acute unloading and gene mitochondrial and Ca^2+^ gene regulation

### Presented by Lija Swain, PhD

Dr. Swain presented her findings on how mechanical unloading of the left ventricle (LV) preserves myocardial mitochondrial structure and function in acute myocardial infarction (AMI). In the past, Dr. Kapur’s research group published in pre-clinical models that unloading the LV using Impella, a transvalvular pump before reperfusion (Primary unloading) reduces LV wall stress and infarct size compared to primary reperfusion [9]. In addition, in a recent study, they demonstrated that primary unloading activates cardioprotective cell signaling pathways that promote cell survival post-myocardial infarction [10]. Through the use of genomic approaches, the data presented by Dr. Swain at the A-CURE Symposium identified mitochondrial integrity/function as a potentially important mechanism by which primary unloading limits myocardial damage.

Interestingly, prior work by Esposito et al. showed that primary unloading preserves mitochondrial structure [10]. To further test and confirm this effect of LV load on mitochondrial function, adult male swine were subjected to left anterior descending artery (LAD) occlusion for 90 min followed by either immediate reperfusion (Primary Reperfusion); LV unloading for 30 min with an Impella CP and then reperfusion (Impella Group), or LV unloading for 30 min with VA-ECMO and then reperfusion (ECMO Group). Using RT-PCR, Dr. Swain confirmed that the expression of genes associated with electron transport chain (ETC) complexes were preserved within the infarct zone of Impella-treated pigs alone and not in ischemia-reperfusion injury or VA-ECMO treated pigs. Her data indicate that this gene regulation effect occurs simultaneously with primary unloading, suggesting an early effect of unloading on gene regulation post-MI.

Using the Agilent Seahorse Platform, mitochondria were isolated from the infarct zone of pigs subjected to the 3 different conditions, and oxygen consumption was quantified in response to various agonist and antagonists of the ETC complexes. The preliminary results suggest that compared to the primary reperfusion or ECMO groups, primary unloading using Impella CP preserves the function of Complex 1 in AMI.

In summary, these findings suggest that primary unloading using Impella may preserve mitochondrial structure and function in AMI. Future studies will confirm these findings and test novel approaches to protect mitochondrial function in AMI.

## Automated Impella maximum unloading system (AIMUS): Exploration and development of first generation autopilot system (smart Impella system)

### Presented by Kenji Sunagawa, MD, PhD

Dr. Sunagawa‘s talk focused on his investigations to develop an algorithm to automate control of the Impella (Smart-Impella) to maximally unload the left ventricle (LV). He reminded the audience that the left ventricular (LV) mechanical work and heart rate (HR) are major determinants of myocardial oxygen consumption (MVO_2_) [11]. Also, the LV pressure-volume area (PVA) represents the total mechanical work of the heart and correlates linearly with MVO_2_ [12]. Recent studies from his research group have shown that total support by Impella, in which the LV no longer ejects blood and the aortic valve remains closed, significantly reduces PVA and, subsequently, MVO_2_ [13–15]. This reduction in MVO_2_ results in a markedly reduced infarct size.

However, hemodynamics change with unloading conditions. In particular, Dr. Sunagawa focused on how the speed of the Impella pump, that is the level of mechanical support provided, impacted the unloading conditions. Based on the data from his studies, under total support, a little change in Impella speed results in a major change in LV systolic pressure (1 mmHg/50 rpm). Such a small change in rpm is difficult to achieve via manual control of the Impella and thus makes its clinical application impractical. To overcome this issue, Dr. Sunagawa’s team developed a Smart-Impella algorithm that controls the instantaneous speed of Impella by incorporating left ventricular pressure and aortic pressure in feedback loops and evaluated its performance in a dog model of acute myocardial infarction (AMI).

Briefly, in 8 dogs AMI was induced for 180 min followed by reperfusion. The Smart-Impella algorithm was introduced 60 min after ischemia and continued for 60 min after reperfusion (I/R). The LV function and infarct size among dogs treated with or without Smart-Impella (*n* = 4 each) was evaluated four weeks after I/R. The results showed that the Smart-Impella treated dogs had nearly normalized LV end-diastolic pressure, LV end-systolic elastance, serum NT pro-BNP level and markedly reduced infarct size more than 60% compared to control. Future studies will focus on potential application of this automated control system to other parameters such as regulation of heart rate or oxygenation.

Dr. Sunagawa also presented the results showing combined use of ECMO and total Impella support during AMI significantly reduced infarct size via marked suppression of MVO_2_. He concluded that these findings demonstrate that Smart-Impella is capable of auto-piloting LV unloading and reducing infarct size. Clinical translation of Smart-Impella combined with education may help improve the quality of care in medicine.

## Mechanical circulatory support by VA ECMO or Impella CP and impact on left ventricular unloading and end organ perfusion in a porcine model of profound cardiogenic shock

### Presented by Jacob Møller, MD, PhD, DMSc

Dr. Møller commenced his talk by stating that as a clinical cardiologist it is very important for him to understand the evolving technologies and the whole concept of unloading the heart in a clinical setting, particularly in patients with cardiogenic shock (CS). Since it is close to impossible to conduct controlled hemodynamic studies evaluating the effect of mechanical circulatory support (MCS) in patients with CS, his research team has developed an animal model that mimics CS after myocardial infarction (MI). This model allows his team to acquire a detailed assessment of hemodynamics, while mimicking human anatomy which allows for the study of the placement of percutaneous MCS devices.

In an earlier study from Norway, investigators were able induce left ventricular (LV) failure by serial injections of 50 μm microspheres into the coronary artery. By doing so, they were able to titrate the degree of LV failure and CS [16]. Dr. Møller research team adopted this approach into their pig model and were able to induce profound CS by injecting microspheres stepwise into the left main coronary artery [17]. Profound CS was defined as cardiac output ≤2 L/min, mean arterial pressure (MAP) of about 30 mmHg, lactate of about 4 mmol/L, and/or a mixed venous saturation (SvO_2_) ≤ 35%. Of note, his lab has been successful in establishing this model in pigs without losing a single animal. Using this model, they compared two MCS devices, the Impella CP and VA-ECMO, and their relative effect on LV end-diastolic pressure (LVEDP) and end-organ perfusion in 12 pigs. Immediately following implantation of both MCS device, cardiac output and SvO_2_ normalized and the devices performed as expected.

**Fig. 2:**
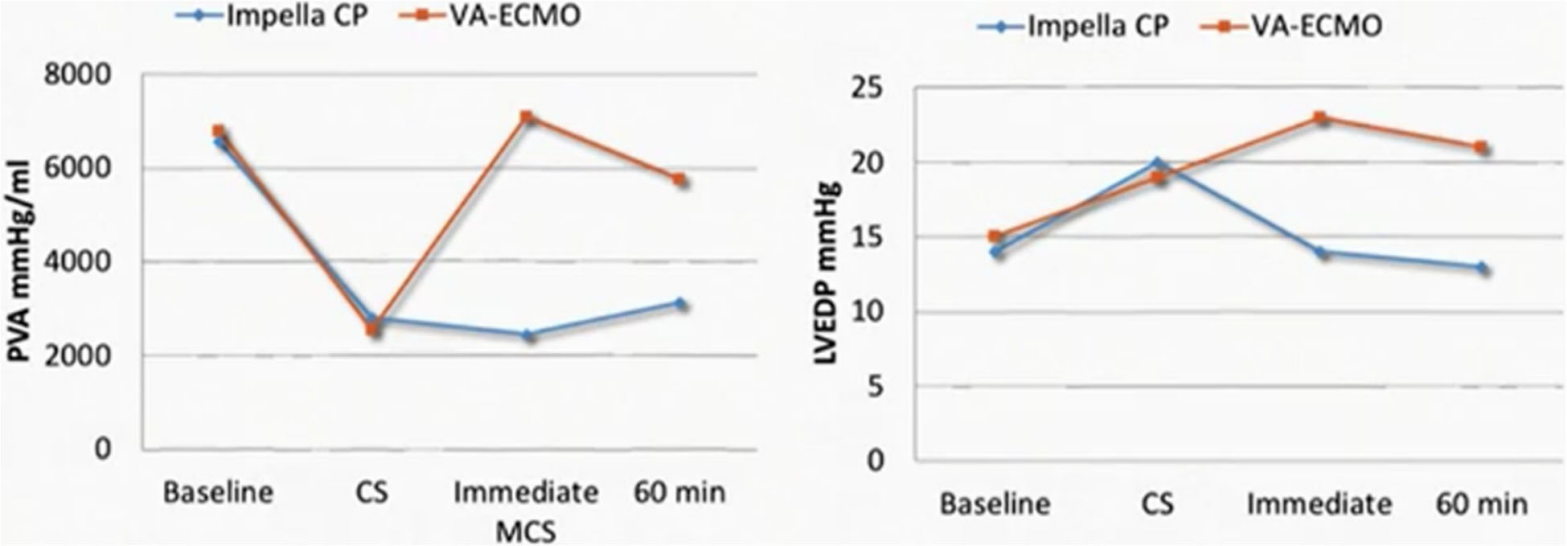
Effect of MCS Devices on PVA and LVEDP in an Animal Model of Profound Cardiogenic Shock. CS = cardiogenic shock; MCS = mechanical circulatory support; PVA = pressure-volume area; LVEDP = left ventricular end-diastolic pressure; VA-ECMO = veno-arterial extracorporeal membrane oxygenation

The hemodynamic consequences of these devices diverged (Fig. 2). Treatment with Impella CP resulted in an immediate decrease in LV end-diastolic volume (LVEDV) and LVEDP. This significantly diminished the pressure-volume area (PVA), a measure of the mechanical work of the heart. In contrast, treatment with VA-ECMO resulted in immediate increase in LVEDV, LVEDP, and PVA, indicating increased mechanical work being conducted by the heart while on ECMO support. While the immediate impact of ECMO on PVA partially normalized after 1 h of support, PVA remained significantly elevated. This contrasted Impella-supported hearts in which PVA was immediately and significantly decreased, and this effected was maintained for the entire duration of support. This data supports the hypothesis that Impella CP efficiently unloads the heart compared to VA-ECMO during CS.

Based on these findings, Dr. Møller proposed as a next step to investigate the effect of different types of vasopressors in combination with Impella CP and titrate the dose optimally.

## Left ventricular unloading with Impella causes cardiac metabolic remodeling with a significant reduction in myocardial glucose consumption and lactate production

### Presented by Carlos Santos-Gallego, MD

Dr. Santos-Gallego presented results from his investigation into how left ventricular unloading using Impella may alter the metabolic remodeling in a model of sub-acute heart failure (HF). He began by providing the background of cardiac metabolism. Normal myocardium produces ATP mainly through free fatty acids (FFA) oxidation [18]. Any abnormalities in myocardial energetics can both cause and contribute to (HF). Since FFA oxidation requires more oxygen, during HF there is a metabolic switch to glucose consumption (which produces less ATP but also requires less oxygen) [18]. Recent studies have demonstrated that this increase in glucose consumption during HF is metabolized mostly through the anaerobic respiration pathway and results in increased production of lactate [19].

Dr. Santos-Gallego investigated the effects of LV unloading and overloading on myocardial metabolism. He hypothesized that acute LV unloading using Impella would reduce myocardial glucose consumption and lactate production, thus reversing adverse metabolic remodeling. He measured the transmyocardial gradient (TG) of different metabolites using simultaneous catheterization of left anterior descending (LAD) and coronary sinus in pigs. Three experimental groups were compared: Sham animals with no myocardial infarction (MI), 1 week post-MI with acute unloading with Impella (before and 2 h after unloading), and 1 week post-MI with LV overloading due to severe aortic regurgitation (before and 2 h after overloading).

**Fig. 3:**
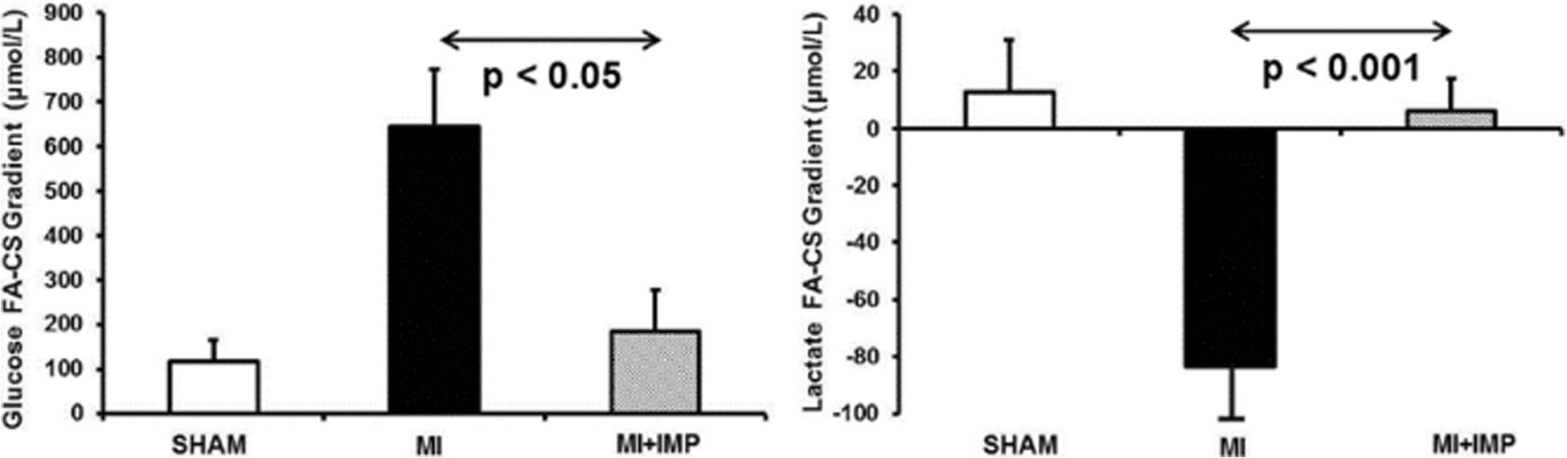
LV Unloading With Impella During HF Reduces Myocardial Glucose Consumption and Lactate Production in an Animal Model. MI = myocardial infarction; IMP = Impella

Results showed FFA consumption with low utilization of glucose and only mild lactate consumption in sham animals (Fig. 3). However, animals in HF showed reduced FFA consumption with increased glucose utilization and lactate production. LV overloading further exacerbated myocardial glucose consumption and lactate production. Contrarily, LV unloading reduced both glucose consumption and lactate production. No difference in myocardial uptake of FFA or ketones was found during LV unloading or LV overloading. These results support the hypothesis that LV unloading with Impella in HF rapidly shifts metabolic substrate utilization with reduced glucose consumption and lactate production.

## Primary left ventricular unloading enhances collateral blood flow and reduces infarct size: A preclinical proof of concept study

### Presented by Shiva Annamalai, MD

Dr. Annamalai began his presentation by recapping the findings of Kapur lab over the years that have demonstrated reduced infarct size with primary unloading prior to reperfusion, irrespective of the MCS device [9, 20]. One of the mechanisms of beneficial effect of primary unloading with Impella is the reduction in LV stroke work, thereby resulting in reduced myocardial oxygen consumption [21]. Another proposed mechanism is the functional reperfusion of the area at risk with Impella support. Dr. Annamalai proposed that the activation of a trans-valvular pump increases myocardial perfusion into the ischemic territory, despite persistent coronary occlusion. By increasing blood supply into the ischemic zone, this should translate into decreased cell death and a smaller infarct size. This concept was demonstrated by Smalling and Wampler in 1989 in a canine model, wherein use of a Hemopump resulted in improved perfusion into the ischemic territory despite persistent occlusion of left anterior descending artery (LAD) [22]. This improved myocardial perfusion to ischemic territory is likely due to improved collateral flow.

The collateral flow index (CFI) is a measure of microcirculatory blood flow and increased CFI has previously been shown to be a primary determinant of myocardial infarct size [23, 24]. Based on previous observations, Dr. Annamalai hypothesized that compared to primary reperfusion, primary unloading recruits collateral microcirculatory flow, thereby increasing ‘functional perfusion’ to the ischemic zone and decreasing infarct size. To test his hypothesis, he assessed CFI in a porcine model of acute myocardial infarction (AMI). The LAD was occluded for 90 min in adult Yorkshire swine (*n* = 4). Then the LAD was reperfused for 180 min in the primary reperfusion (PR) group. In the primary unloading (PU) group an Impella CP and in primary loading (PL) group, VA ECMO, was activated with LAD occlusion for additional 30 min, followed by 180 min of reperfusion. The CFI was calculated during LAD occlusion as (Pw-RA)/(Pa-RA), where Pa, RA, Pw are aortic, right atrial and coronary wedge pressure, measured using a pressure wire. The myocardial infarct size was quantified using 2,3,5-triphenyltetrazolium (TTC) staining.

**Fig. 4:**
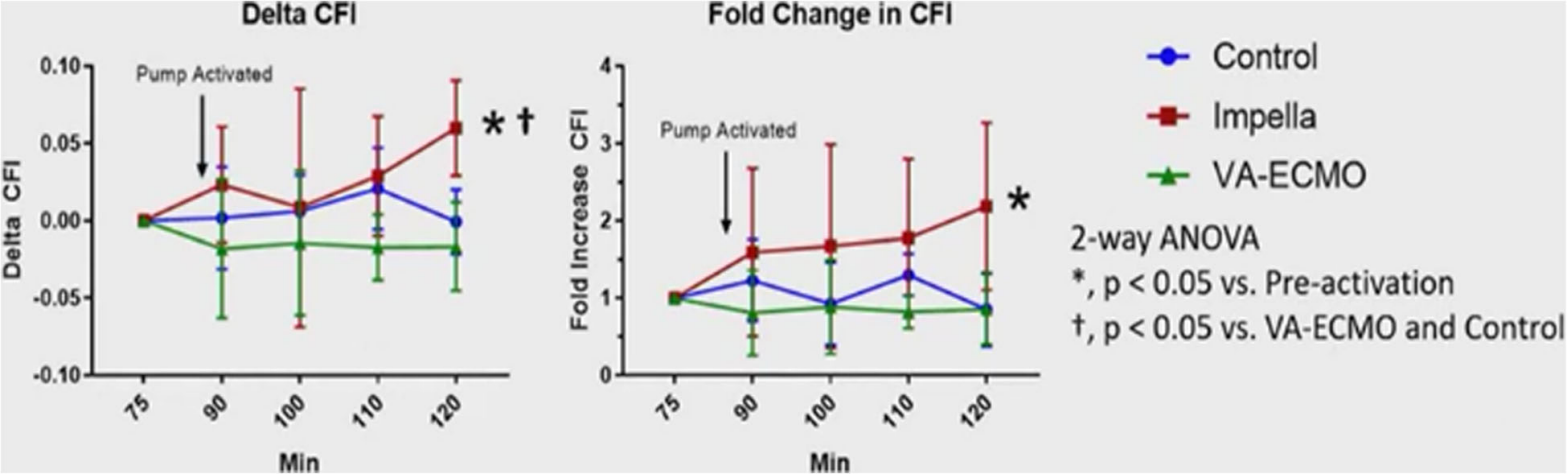
LV Unloading With Impella for 30 min Increases Microcirculatory Flow to the Area at Risk. CFI = collateral flow index; VA-ECMO = veno-arterial extracorporeal membrane oxygenation

Results showed that following 90 min of LAD occlusion, there was no difference in CFI among the groups as expected. However, after 30 min of PU, the CFI significantly increased compared to pre-activation as well as to the PR or PL groups. This suggests increased microcirculatory flow to the area at risk with 30 min of LV unloading with Impella prior to reperfusion (Fig. 4). In addition, the hemodynamic tracings showed a rise in distal coronary wedge pressure with Impella, likely contributing to increase in CFI. PU also reduced LV stroke work (LVSW) at 120 min compared to each of pre-activation, PR and PL. Among all the groups, the change in CFI between 90 and 120 min correlated inversely with the change in LVSW. Compared to PR and PL, PU reduced infarct size relative to the area at risk, and the change in CFI correlated inversely with infarct size.

In conclusion, this is the first study to show that reducing LVSW with Impella prior to reperfusion increases collateral flow to the infarct related artery in acute myocardial infarction. In contrast to Impella, activation of VA-ECMO does not augment collateral flow and does not reduce infarct size.

## Keynote lecture

### Left ventricular remodeling: Therapeutic effects, implications for trials and lessons from chronic heart failure

#### Presented by James Udelson, MD

Dr. Udelson‘s talk focused on his research interest in left ventricular remodeling and the lessons learned from clinical trials in the field of chronic heart failure. He began by highlighting the remarkable plasticity of the heart with the ability to grow larger by 50% during hypertrophy and shrink by 30% during atrophy [25]. Physiologic remodeling of the heart occurs in response to increased demand on cardiac output such as is associated with exercise training and pregnancy, and reverse remodeling occurs after birth or during deconditioning. Pathologic remodeling with hypertrophic growth occurs in response to hemodynamic stress such as hypertension or myocardial injury, and it increases the risk of heart failure (HF) [25]. Hence, the focus of many research teams over the years has been to understand the mechanisms of pathological remodeling following myocardial infarction (MI).

Early work by Pfeffer in 1991 demonstrated the schematics of LV volume change post-MI in a rat model [26]. During the early post infarction phase, there is infarct expansion but then the remaining normal myocardium undergoes hypertrophy and chamber dilation. This is initially an adaptive response to maintain stroke volume and hemodynamics. However, continuous unabated hypertrophy and dilation over time is maladaptive as cardiac function declines with increased dilation and volume overload. The pathological process of progressive ventricular dilation post-MI was demonstrated in humans as well by McKay and colleagues [27].

Later studies investigated the relation between post-MI remodeling and the incidence of cardiovascular adverse events. Results from the secondary analysis of SAVE trial showed higher rates of adverse cardiovascular events with greater increase in LV enlargement, independent of randomization to captopril [angiotensin-converting enzyme (ACE) inhibitor] or placebo [28]. Subsequent studies also demonstrated that the change in LV shape, size, volume, and architecture post-MI relates to subsequent adverse cardiovascular events.

Given the high rate of adverse events associated with ventricular remodeling post-MI, research efforts have focused on assessing the effect of drugs and devices in attenuating ventricular remodeling. Early work by Pfeffer et al. in a rat model showed that captopril therapy for 3 months post-MI attenuated LV remodeling and the deterioration of cardiac performance in chronic MI [29]. Further, they showed that long-term therapy with captopril also improved 1-year survival [29]. Consistent with this finding, clinical data from the SAVE trial demonstrated a reduction in mortality with in patients with LV dysfunction after MI who were treated with captopril [30].

Results from additional trials with ACE inhibitors and beta-blocker therapy post-MI and in HF, including the SOLVD and CAPRICORN trials, suggested a pattern of reduced LV volume and concomitant decrease in mortality. Along the same lines, the PRIME-II trial that investigated the use of the dopaminergic receptor agonist, ibopamine, in patients with chronic HF also showed increased LV volumes to be associated with increased mortality. These findings led to the attractive supposition that LV remodeling was a fundamental feature of the post-MI or CHF disease process and progression, and the effect of the intervention on remodeling may act as a “surrogate” for its potential impact on outcome [31]. However, the RENEWAL trial with etanercept showed a decrease in LV volume with no difference in the mortality rates, thus questioning the use of variables of ventricular remodeling as a surrogate for patient outcomes.

Dr. Udelson discussed the use of biomarkers as potential surrogates for patient outcomes. Fleming and DeMets define a biomarker as a strong surrogate if: 1) it is in the causal path between an intervention and the outcome; 2) all intervention effects pass through the marker in the causal pathway; and most importantly 3), the effect of the intervention on the surrogate reliably predicts the overall effect on the clinical outcome [32]. Of note, sample sizes of recent clinical trials assessing the outcome of new HF drugs range from 3 to 8000 patients. This is mainly due to low event rates of the primary endpoint making the number of patients needed to adequately power the study to detect a clinically meaningful difference. This leads to long and costly clinical trials. Consequently, there is a lot of interest in identifying biomarkers surrogates in phase 2 clinical trials (involving few hundreds of patients) that can predict success or failure of phase 3 clinical trials (involving 1000 of patients).

However, results from the CAST and ILLUMINATE trial have demonstrated that biomarkers that are prognostic are not necessarily good surrogates. Premature ventricular contractions (PVCs) are known to associated with unfavorable prognosis post-MI; however, in CAST trial suppression of PVCs was associated with increased mortality. Similarly, ILLUMINATE demonstrated that increased HDL was associated with increased mortality, counter to prevailing thought. Dr. Udelson cautioned that this danger was not just limited to biomarker use. Even successful outcomes in a phase 2 trial don’t necessarily predict successful outcomes in phase 3 trials. This was demonstrated by the opposite effect of vesnarinone on mortality in the phase 3 VEST trial compared to the preceding phase 2 trial. Since heterogenous pathways contribute to the progression of HF and resulting mortality, it is unlikely that any individual marker will be able to predict the clinical outcome of interest with precision. However, the magnitude of correlation of an intervention on a biomarker, and the effect of that intervention on longer-term outcomes should be quantifiable. Therefore, Dr. Udelson suggested that the change in a biomarker following an intervention should be viewed as a ‘probability signal’ of outcome effects, rather than as a precise surrogate. He highlighted the study by Kramer et al. that demonstrated a positive correlation between an intervention’s short-term effect on LV remodeling and long-term mortality in HF. This study concluded that the effect of interventions on LV remodeling can serve as a probability signal of the likelihood of a favorable, neutral, or adverse effect on long-term mortality [33].

Dr. Udelson discussed strategies used by contemporary clinical trials to overcome the challenges such as huge sample size and contribution of only 10–15% of patients enrolled in the trial to the endpoint. He mentioned the use of hierarchical composites such as such as the Finkelstein-Schoenfeld method, and other analytic approaches including adaptive design and expedited access pathway to assess the outcome of an intervention using a reasonable sample size.

In conclusion, LV remodeling is a stereotypical response to injury underlying pathology. Remodeling is associated with adverse outcomes and is a fundamental driver of HF progression. The remodeling response to an intervention provides a probability signal of the intervention’s response to the clinical outcome, and this concept can be leveraged into novel trial designs to enhance the likelihood of outcome response with smaller sample sizes.

## The cardio-renal system: Acute kidney injury and MCS, opportunity for improved patient outcomes

### Presented by Ralf Westenfeld, MD

Dr. Westenfeld began by referring to the recent PRESERVE trial showing a lack of benefit of bicarbonate and acetylcysteine for prevention of contrast-induced acute kidney injury (CI-AKI) [34]. This highlighted the unmet medical need to address CI-AKI in patients treated by percutaneous intervention (PCI) as it is associated with high mortality and increases mortality in patients with chronic kidney disease (CKD) [35]. In addition, CI-AKI ranks second among the causes of AKI [36]. An aversion to the risk of radiocontrast-associated nephrotoxicity has resulted in the less frequent use of coronary angiography among patients with CKD despite significant benefit in the odds of survival, also referred to as “renalism” [37].

The pathophysiology of CI-AKI is related to the decline in glomerular filtration rate (GFR) caused by renal vasoconstriction, oxidative stress due to reduced renal blood flow, and renal tubular cell damage. Many interventions aimed at preventing CI-AKI have been tested but the protective effect has been observed only in studies of low quality, and no effect of the intervention was observed in high quality studies. According to a recent publication by Vanmassenhove et al., only volume loading for prevention of CI-AKI has proven to be of value [38]. Dr. Westenfeld further discussed the pros and cons of different biomarkers and imaging techniques used to detect or foresee AKI.

**Fig. 5:**
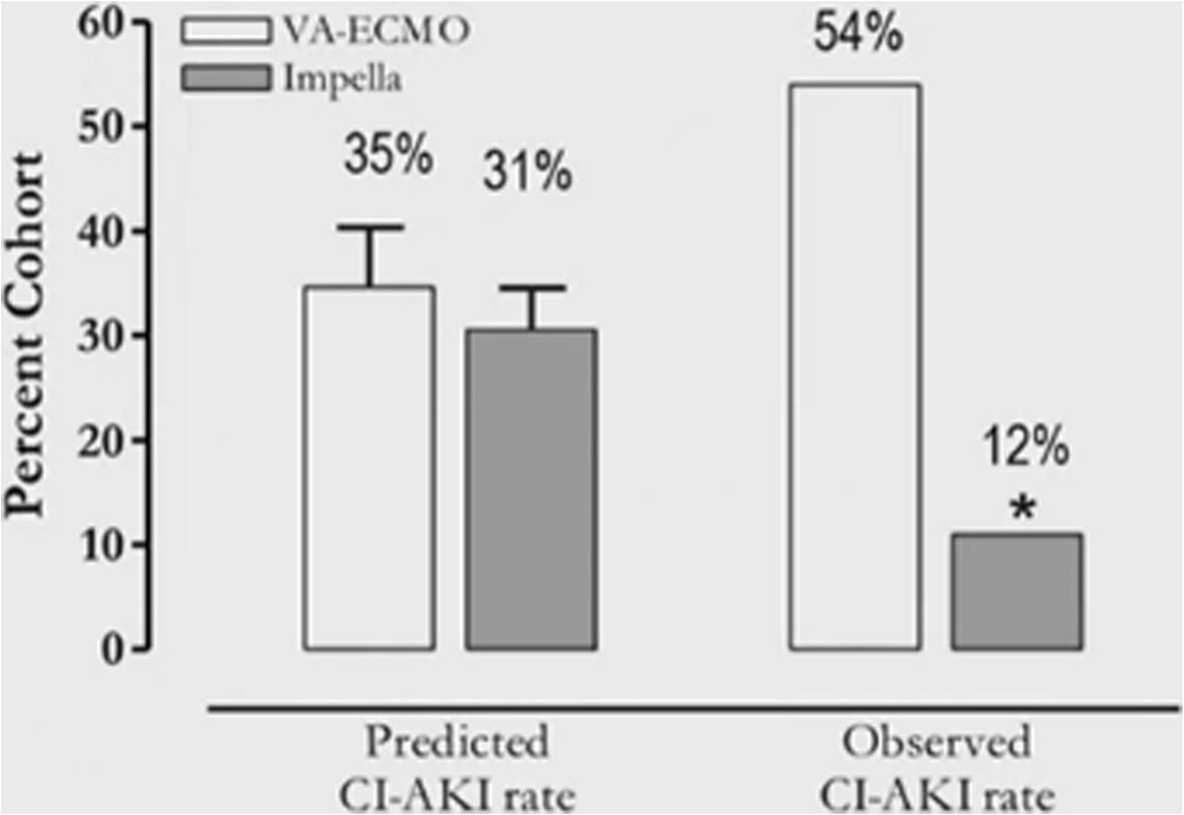
Lower Incidence of CI-AKI During HR-PCI with Impella support versus VA-ECMO. CI-AKI = contrast induced acute kidney injury; HR-PCI = high risk percutaneous coronary intervention, VA-ECMO = veno-arterial extracorporeal membrane oxygenation

A recent study by Flaherty et al. was the first to demonstrate protection against CI-AKI by Impella 2.5. Interestingly, Impella 2.5 support was independently associated with a significant reduction in the risk of developing AKI during high-risk percutaneous coronary intervention (HRPCI) [39]. This led Dr. Westenfeld to question whether other mechanical circulatory support devices may be beneficial in preventing AKI during HRPCI. His research team investigated this by performing a retrospective analysis of 28 patients undergoing HRPCI with the support of Impella versus VA-ECMO. The results were consistent with the study Flaherty et al. with a lower incidence of CI-AKI in patients supported by the Impella versus VA-ECMO (12% vs. 54%, *p* < 0.05) (Fig. 5). In addition, they observed that with Impella support equally complex coronary interventions could be performed with shorter time and less bleeding events.

Dr. Westenfeld concluded by proposing a prospective “Protect Kidney Trial” to investigate the role of Impella in preventing CI-AKI in patients undergoing HRPCI.

## Biomarkers of kidney injury in a preclinical model of acute myocardial infarction

### Presented by Navin Kapur, MD on behalf of Xiaoying Qiao, PhD

Cardiorenal syndrome type 1 refers to the condition of acutely decompensated heart failure (ADHF) leading to kidney dysfunction. During heart failure (HF) the presence of venous congestion can increase renal vein pressure and interstitial pressure which compresses Bowman’s capsule leading to a reduced glomerular filtration rate (GFR). A recent study from Ichiki et al. showed significant upregulation of several inflammatory cytokines, particularly in the renal cortex, in a canine model of HF, providing insights into the pathophysiology of kidney dysfunction in HF [40].

Since several hemodynamic parameters influence kidney function, Dr. Kapur’s research team is investigating the effect of acute mechanical circulatory support devices on renal blood flow and function. Previously, Møller-Helgestad et al. compared the hemodynamics and blood flow to the kidneys with intraaortic balloon pump (IABP) versus Impella 2.5. They found higher renal blood flow (RBF) with Impella 2.5 and no change with IABP support [41]. Studies have reported up to 60% incidence of acute kidney injury (AKI) with VA-ECMO due to mechanisms such as nonpulsatile flow and inflammatory response resulting in reduced RBF [42, 43].

Based on these findings, Dr. Qiao hypothesized that compared to Impella, VA-ECMO would increase levels of renal injury biomarkers in acute myocardial infarction. To test this hypothesis, adult male swine were subjected to left anterior descending artery (LAD) occlusion for 90 min followed by either immediate reperfusion (IRI), ventricular unloading with Impella for 30 min prior to reperfusion while on support, VA-ECMO support for 30 min prior reperfusion while on support, or sham-operated controls (*n* = 4/group). Renal injury biomarkers, kidney injury molecular 1 (KIM1) and NGAL, were measured. Results showed that the urinary KIM1 levels were elevated in the IRI and VA-ECMO groups, but not the Impella group. No changes in plasma KIM-1 levels were observed in any group. Compared to baseline values, VA-ECMO increased urinary NGAL levels but Impella did not. Compared to IRI, Impella reduced plasma NGAL levels after reperfusion. Further, Impella reduced urinary Cystatin-C (Cys-C) levels from the baseline but VA-ECMO did not. No change in plasma Cys-C levels was observed between groups.

In conclusion, this is the first study to identify IRI increases increases urinary levels of KIM-1, a highly sensitive biomarker of AKI. Impella, not VA-ECMO, may protect against this increase in urinary KIM-1 levels. Future studies will explore the mechanism underlying this observation.

## Impella is associated with reduced incidence of contrast-induced nephropathy in patients undergoing high risk PCI

### Presented by Julian Wiora, MD

Dr. Wiora presented results from his investigation into the effect of Impella support on contrast-induced acute kidney injury (CI-AKI) in patients undergoing high-risk percutaneous coronary intervention (HR-PCI). He reminded the audience that approximately 7% of all patients undergoing PCI experience AKI regardless of comorbidities [44], and the incidence of AKI and need for dialysis after PCI increases significantly with increasing severity of baseline chronic kidney disease (glomerular filtration rate, GFR < 30 ml/min/1.73 m^2^).

A recent study by Flaherty et al. was the first to demonstrate protection against CI-AKI by Impella 2.5 compared to no support in high-risk PCI patients. Interestingly, Impella 2.5 support was independently associated with a significant reduction in the risk of developing AKI during high-risk PCI [39]. Dr. Wiora performed a similar analysis by comparing 35 patients who underwent HR-PCI with Impella support to 35 patients who underwent PCI without indication for Impella support in their institution. The patients in the two cohorts were propensity-matched for risk of contrast-induced nephropathy based on the Mehran risk score.

**Fig. 6:**
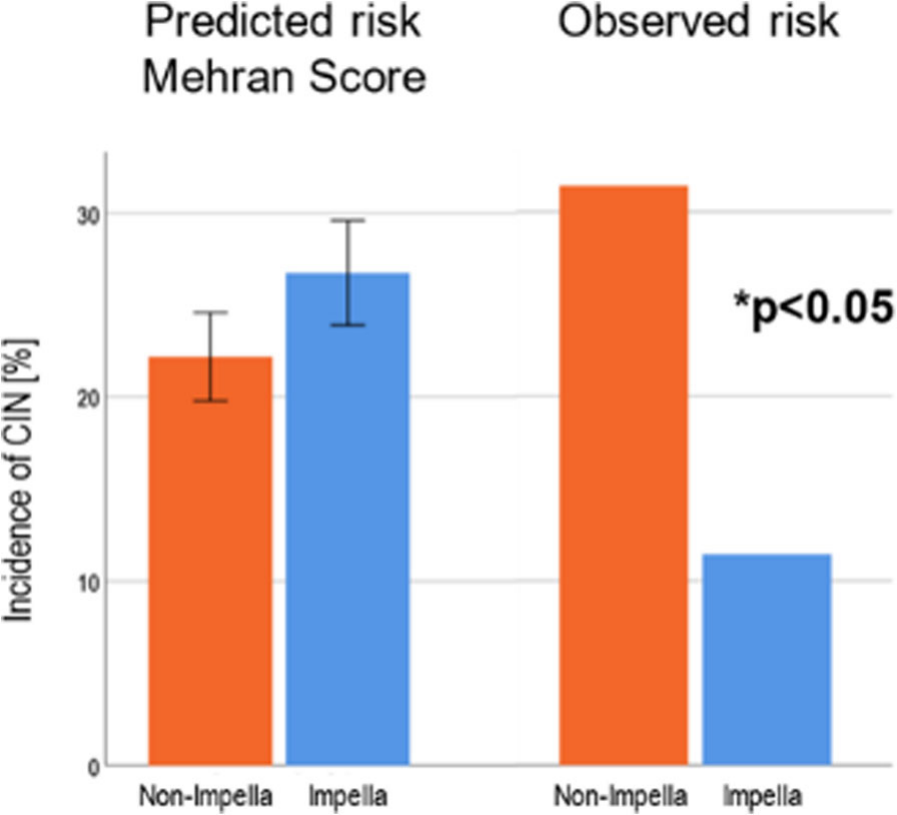
Reduced Incidence of Contrast-Induced Nephropathy with Impella Support during HR-PCI. CIN = Contrast-Induced Nephropathy

Patients in the non-Impella support group were older and had less compromised left ventricular ejection fraction (LVEF) than patients in the Impella support group. In the Impella group, the procedure time was longer with more number of lesions treated and stents implanted than the non-Impella group. Based on the Mehran risk score, CIN rates were predicted to be 25% with Impella support and 22% without Impella support. Interestingly, CIN was observed in only 11% of patients undergoing Impella-protected PCI compared to 31% in the non-Impella supported group (Fig. 6). In addition, serum creatinine levels were significantly lower in the Impella group over 4 days post-PCI. Furthermore, among the patients in the non-Impella group, CIN occurred more frequently in patients with lower LVEF and hemoglobin levels, but no such trends were observed in the Impella supported group. No difference in complication rates were observed between groups.

In conclusion, Impella use during HR-PCI was associated with a lower incidence of CIN compared to a propensity-matched unprotected control group. Use of Impella during HR-PCI is safe and feasible based on the low complications rates. Future studies will focus on the use of Impella during HR-PCI in patients with moderately reduced LVEF.

## Impact of unloading during AMI-CGS: The Hannover cardiac unloading registry (HACURE) and European multicenter experience

### Presented by Andreas Schäfer

Dr. Schäfer began by stating that acute myocardial infarction complicated by cardiogenic shock (AMICS) is a growing clinical challenge in clinical practice. Conventional therapeutic approaches using inotropic support with or without intra-aortic balloon pump have not improved outcomes. Retrospective studies have associated early identification and standardized treatment including early initiation of hemodynamic support with Impella followed by weaning of inotropes and complete revascularization with reduced mortality. Given the challenges of conducting randomized trials in CS and the lack of evidence on the efficacy of Impella from prospective trials thereof, Dr. Schäfer presented results from a retrospective analysis conducted by his research team in a contemporary cohort.

First, he highlighted the recently published Hannover Cardiac Resuscitation Algorithm (HaCRA) for patients presenting with Out-of-hospital cardiac arrest (OHCA) and/or cardiogenic shock [45]. The algorithm aims for early diagnosis and prompt treatment of life-threatening conditions such as cardiogenic shock. He emphasized the need to intervene using mechanical circulatory support (MCS) devices early during the “golden hour” of cardiogenic shock and not during later stages when multiorgan dysfunction and failure has already begun.

He then referred to a recent publication of 61 patients with cardiogenic shock (CS) supported using Impella CP for isolated left ventricular (LV) failure in the HAnnover Cardiac Unloading REgistry (HACURE) [46]. Comparison of survivors versus non-survivors showed that survivors had pronounced lactate clearance within 4 h of Impella initiation. This was also accompanied by a rapid reduction in vasopressors/inotropes. Importantly, a substantial reduction in 30-day mortality among the subgroup of patients (*n* = 25) fulfilling the inclusion/exclusion criteria of the former IABP-Shock II trial was observed (24% in HACURE using Impella CP vs. 40% in IABP-Shock II trial), highlighting the importance of a standardized algorithm for treatment of CS incorporating early use of active hemodynamic support using Impella.

He then presented the retrospective analysis of > 160 patients with AMICS treated at four dedicated European shock centers, fulfilling the inclusion criteria of the IABP-Shock II trial, mostly treated with Impella CP. Risk of mortality was calculated based on IABP-Shock II, CardShock, and SAVE scores. Cardiac arrest prior to Impella implantation was relatively common. Impella was implanted prior to percutaneous coronary intervention (PCI) due to the operator’s discretion. While overall, 30-day mortality for the entire cohort was still slightly above 40%, mortality was higher among patients undergoing resuscitation and among those receiving Impella post-PCI. The predicted in-hospital mortality was 69% by SAVE score, 40% by CardShock score, and 49% by IABP-Shock II score, respectively. The observed 30-day mortality with use of Impella devices was lower than the predicted mortality. Furthermore, the observed mortality was lower than predicted mortality even among patients in the high-risk subgroup.

In conclusion, the results from these retrospective analyses encourage the use of active MCS by Impella devices in critically-ill patients with AMICS undergoing PCI and support the concept of early initiation of support prior to PCI.

## First in man implantations of a newly designed transaortic axial flow ventricular assist device, Impella 5.5

### Presented by Alexander Bernhardt, MD

Dr. Bernhardt began by reminding the audience of the features of the Impella 5.0. This device is an established transaortic axial flow ventricular assist device capable of providing forward blood flow of up to 5 L/min. It was originally designed for femoral access but axillary access is increasingly used as it allows for mobilization of the patient. Recently, a published meta-analysis of Impella 5.0 reported favorable survival outcomes and high rates of myocardial recovery in patients with cardiogenic shock [47]. Impella 5.0 has CE approval for a maximum to 10 days of support. However, more than 70% of patients at their hospital require Impella 5.0 support for > 10 days. Potential problems associated with longer duration of Impella 5.0 support include in-growth of the pig tail catheter, pump thrombosis, and arterial embolization due to the presence of a repositioning sheath in the axillary artery.

**Fig. 7:**
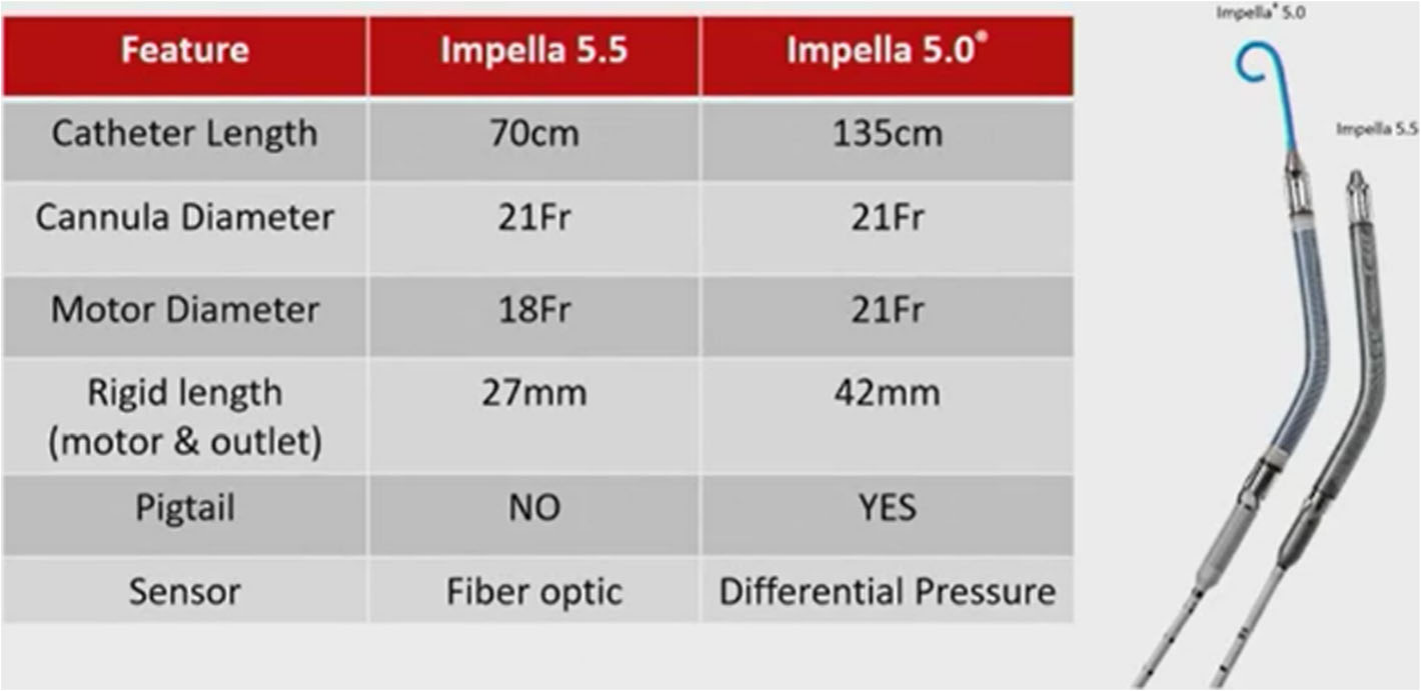
Summary of Features of Impella 5.5 and Impella 5.0 Heart Pumps

Impella 5.5 was developed to address the need for a longer duration of support with a low rate of complications. It is designed to provide hemodynamic support for up to 30 days. Like the the Impella 5.0, the Impella 5.5 device is an axial flow transaortic cardiac support device mounted on a 9 Fr steering catheter with a 21 Fr pump cannula (Fig. 7). The pump itself is shorter and stiffer than the Impella 5.0. Other improved features in the Impella 5.5 include an optical aortic pressure sensor distal to the outflow of the device and no pigtail at the tip of the catheter (eliminating the risk for in-growth of the pigtail and reducing the risk of thromboembolism and stroke), and improved kink resistance of the cannula. Importantly, the device provides maximum pump flow of 5.5 L/min against a pressure afterload of 60 mmHg, with observed flow up to 5.8 L/min. The device is designed for axillary insertion only and the repositioning sheath does not extend into the artery.

Dr. Bernhardt presented the first-in-man experience with the new Impella 5.5 in two critically ill patients [48]. The first patient was a 75-year-old male with a ten year history of ischemic cardiomyopathy and severe functional mitral regurgitation. The patient was treated with intravenous antibiotics for pneumonia and was on non-invasive ventilation. The left ventricular ejection fraction (LVEF) was 18%, and left ventricular end-diastolic diameter (LVEDD) was 75 mm. His N-terminal pro brain natriuretic peptide (NTproBNP) was 44 pg/ml at baseline. The Impella 5.5 was implanted via left axillary access. The implantation procedure was uneventful and the pulmonary function improved within 24 h. After further improvement in pulmonary congestion and pneumonia, a MitraClip procedure was performed successfully under Impella 5.5 support on post-operative day 10. During follow-up, the patient developed signs of an acute abdominal pain due to an appendicitis. No signs of ischemia or thromboembolism were present. Initially, the patient recovered from this complication, but deceased on post-operative day 19 due to a recurrent septic episode. There were no pump related complications and no signs of a pump malfunction.

The second patient was a 52-year-old male patient with ischemic cardiomyopathy and a history of coronary artery bypass grafting and mitral valve repair. Three weeks prior, the patient suffered an acute decompensation that was treated with Impella CP support for five days. After that episode the patient needed dialysis. He suffered a second decompensation due to ventricular arrhythmia, was resuscitated and placed on a veno-arterial extra corporeal life support (ECLS) through femoral vessel cannulation. The Impella 5.5 was implanted during the same operation. The arrhythmias were treated using catheter ablation under ECLS and Impella 5.5 support, and the patient was extubated. The ECLS cannulas were explanted under local anesthesia the day after the ablation procedure. During Impella 5.5 support, renal function improved and the patient was mobilized. Impella 5.5 support was continued uneventful for 21 days. A durable LVAD (HeartMate 3) was implanted and Impella 5.5 was explanted. The patient is ambulatory at 7 months post-Impella 5.5 implantation with improved creatinine levels and off-dialysis. Two additional patients have been treated using Impella 5.5 since. No pump-related adverse events has been observed.

In conclusion, the Impella 5.5 expands the spectrum of available short-term mechanical circulatory support devices. New technical design features such as the absence of pig-tail helps minimize risk, and the optical pressure sensor aids in easy pump placement and monitoring. Early experience with Impella 5.5 in patients with no occurrence of pump-related adverse events indicate feasibility and safety of the new device.

## Temporal trends and long-term outcomes of post-myocardial infarction heart failure

### Presented by Ajar Kochar, MD, MHS

Dr. Kochar began by presenting the background of heart failure (HF) post-myocardial infarction (MI). Over half of contemporary MI patients are ≥65 years old. Despite decline in MI-related mortality in the past 2 decades, there has been an increase in the incidence of post-MI HF with significant long-term implications. The objective of Dr. Kochar’s research study was to identify the temporal trends and patterns of the incidence of new HF among older MI patients and evaluate the association of post-MI HF with long-term mortality and major adverse cardiac events (MACE).

Dr. Kochar evaluated patients in the Medicare database between 2000 and 2013 who survived their first MI (based on ICD9 coding). Post-MI HF was defined as HF during index MI admission or a HF hospitalization within 1 year post-index MI admission. Outcomes assessed were HF hospitalization and all-cause death at 1 year and all-cause death and MACE (composite of all-cause death, MI and stroke hospitalization) at 5 years. The incidence of post-MI HF was calculated at 1 year based on estimates from the cumulative incidence function. The 5-year implications of HF post-MI was evaluated by comparing patients with HF post-MI with patients without any HF within 1 year post-MI.

**Fig. 8:**
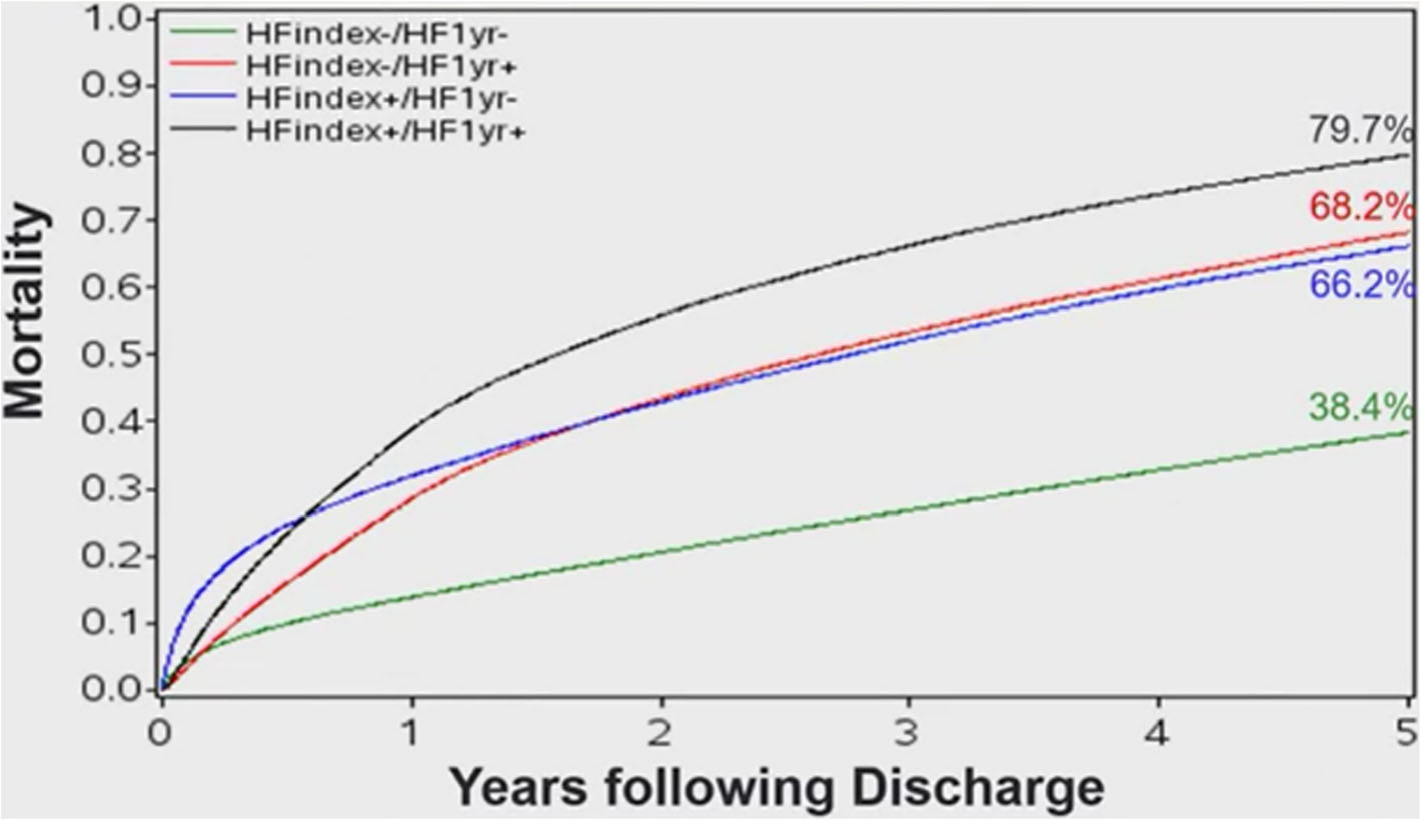
Long-term Mortality Stratified by Heart Failure Status

The study population included 1,531,628 patients from 5948 hospitals. The mean age was 78 years, 49.7% were women, 64.6% had NSTEMI and 35.4% had STEMI. Among these patients, 36% had HF post-MI: 32.8% had HF during index MI and 10.4% had HF hospitalization post-MI within 1 year. The median time to first HF hospitalization was 66 days. The temporal trend from 2000 to 2012 suggests mild reduction in the incidence of HF during index hospitalization (34.7% in 2000 to 31.2% in 2012), HF hospitalization within 1 year (11.3% in 2000 to 8.7% in 2012), and 1-year mortality (22.5% in 2000 to 18.8% in 2012). Five-year mortality was 38.4% for patients without any HF, 66.2% for patients with HF during index admission only, 68.2% for patients with a HF hospitalization within 1 year only, and 79.7% for patients with HF at index admission and at least one HF hospitalization within 1 year (Fig. 8).

Given the high rates of HF post-MI, Dr. Kochar emphasized the importance of optimizing guideline directed therapies in older patients and the need to explore novel treatment dimensions. He added that the high 5-year mortality rates with HF post-MI should be communicated with patients to better inform shared-decision making.

In conclusion, the rate of mortality and HF post-MI has modestly declined over time among older MI patients treated in the US. Despite this decline, post-MI HF remains a common outcome in older adults, occurring in > 1 out of 3 patients. Compared to patients without HF, post-MI HF is associated with higher mortality and MACE.

## Development of heart failure among survivors of STEMI and NSTEMI and long-term outcomes

### Presented by Robert Kloner, MD, PhD

Dr. Kloner presented the results from his investigations aimed to develop a biologic left ventricular pump. He began by providing background for arterial counterpulsation. Arterial counterpulsation is a strategy used as a bridge to transplantation or recovery to treat patients with terminal heart failure. There are 4 different ways for arterial counterpulsation: intra-aortic, extra-aortic, para-aortic and enhanced external counterpulsation. His investigations were focused on determining whether it is feasible to develop a biologic extra-aortic counterpulsation device by cardiac cell implantation. Specifically, his research team tested if immature cardiac cells could be implanted into the outer wall of the aorta in rats, and if so, could they survive, differentiate, and contract [49, 50].

Female Fischer rats received either medium only (*n* = 22) or 5 × 10^6^ neonatal cardiomyocytes each (n = 22). The aorta was exposed through a retroperitoneal approach, and cardiomyocytes isolated from 2 day old neonatal Fischer rats of both sexes were implanted at the outer wall of the abdominal aorta at a site 3 mm above the renal arteries. Histological analysis showed viable graft formation in the outer wall of the aorta at 2 weeks in 9 of 10 rats and at 6 weeks in 9 of 9 rats in the cell-treated group. In comparison, none of the rats in the medium-treated group showed viable graft formation in the outer wall of the aorta. In addition, neonatal cardiomyocytes in the graft formed compact, longitudinally oriented cardiac muscle bundles, and were differentiated with cross-striations and a high degree of vascularization. At 2 weeks after transplantation, spontaneous rhythmic beating was observed at the grafted site following excision of the native heart in 7 out of 10 rats in the cell-treated group versus none in the medium-treated group. Further, pacing in a cell-grafted aorta increased aortic pressure by 5-fold from the baseline.

These results suggest that grafted neonatal cardiomyocytes can survive, differentiate, and develop blood supply with the ability to spontaneously contract within the outer walls of the aorta in the rats. Encouraged by these results, his research team investigated whether it is feasible to develop a vein that rhythmically beats by implanting immature cardiomyocytes in the wall in aorta near the vena cava [51]. Female Fischer rats received either medium only (*n* = 6) or 5 × 10^6^ neonatal cardiomyocytes each (n = 6). The vena cava was exposed through a midline incision of the abdominal wall and cardiomyocytes isolated from 2 days old neonatal Fischer rats of both sexes were implanted around the vena cava below the renal vein.

Histological analysis showed viable graft formation and maturation around the vena cava with cross striations at 3 weeks after transplantation. In addition, following aortic clamping and excision of the native heart, spontaneous rhythmic beating was observed at the grafted site at a rate different than the aortic rate in 100% of the cell-treated group versus none of the medium-treated group. The spontaneous beating rate of vena cava was 101 ± 7 beats at 1–3 min after excision of the native heart. Also, the diameter of the vena cava was reduced by 17.5 ± 5.4% when the grafted cardiac cells contracted. These results show that the grafted neonatal cardiomyocytes around the vena cava can survive, mature, and contract spontaneously.

In conclusion, Dr. Kloner suggested that the potential of cardiac cell transplantation to fashion an external auxiliary circulatory pump should be further investigated.

## Should Door-To-Unload (DTU) replace Door-To-Balloon (DTB) for acute MI without shock

### Presented by Navin Kapur, MD

Dr. Kapur presented the purpose and learnings from the Door-To-Unload (DTU) in ST-elevation in myocardial infarction (STEMI) pilot trial using Impella CP as the mechanical left ventricular unloading platform. He began by providing the background for this trial. By the year 2030, 1 out of every 33 individuals in the United States will have heart failure (HF), and the associated total costs will reach $70 billion per year [52]. For every 5% increase in myocardial infarct size, 1-year HF hospitalization increases by 20% [53]. Additional strategies are therefore needed to reduce myocardial infarct size.

Maroko and Braunwald in 1971 suggested “that measures designed for reduction of myocardial oxygen demands and improvement of coronary perfusion, when effected promptly might reduce the ultimate size of (a myocardial) infarction” [54]. Since 1978, multiple preclinical studies have tested whether reducing myocardial oxygen consumption by implementing a circulatory pump limits myocardial damage in an acute myocardial infarction (AMI). In addition, multiple attempts have been made using pharmacological approaches to reduce myocardial oxygen demand by reducing heart rate, left ventricular (LV) wall stress, and LV stroke work.

Several studies spanning 4 decades have tested mechanical unloading using mechanical devices and demonstrated that unloading before not after reperfusion is required to reduce infarct size [55]. Since 2012, multiple preclinical studies from the Kapur lab have supported the hypothesis that primary LV unloading and delaying coronary reperfusion for 30 min provides both cardioprotective signaling and myocardial salvage [10, 20, 56]. The central hypothesis of the DTU STEMI pilot trial was compared to LV unloading and immediate reperfusion, LV unloading followed by a 30 min delay to reperfusion is feasible and safe as defined by: successful enrollment and protocol completion (feasibility); no increase in major adverse cardiovascular or cerebral events (MACCE, safety); and no increase in infarct size between groups (safety).

**Fig. 9:**
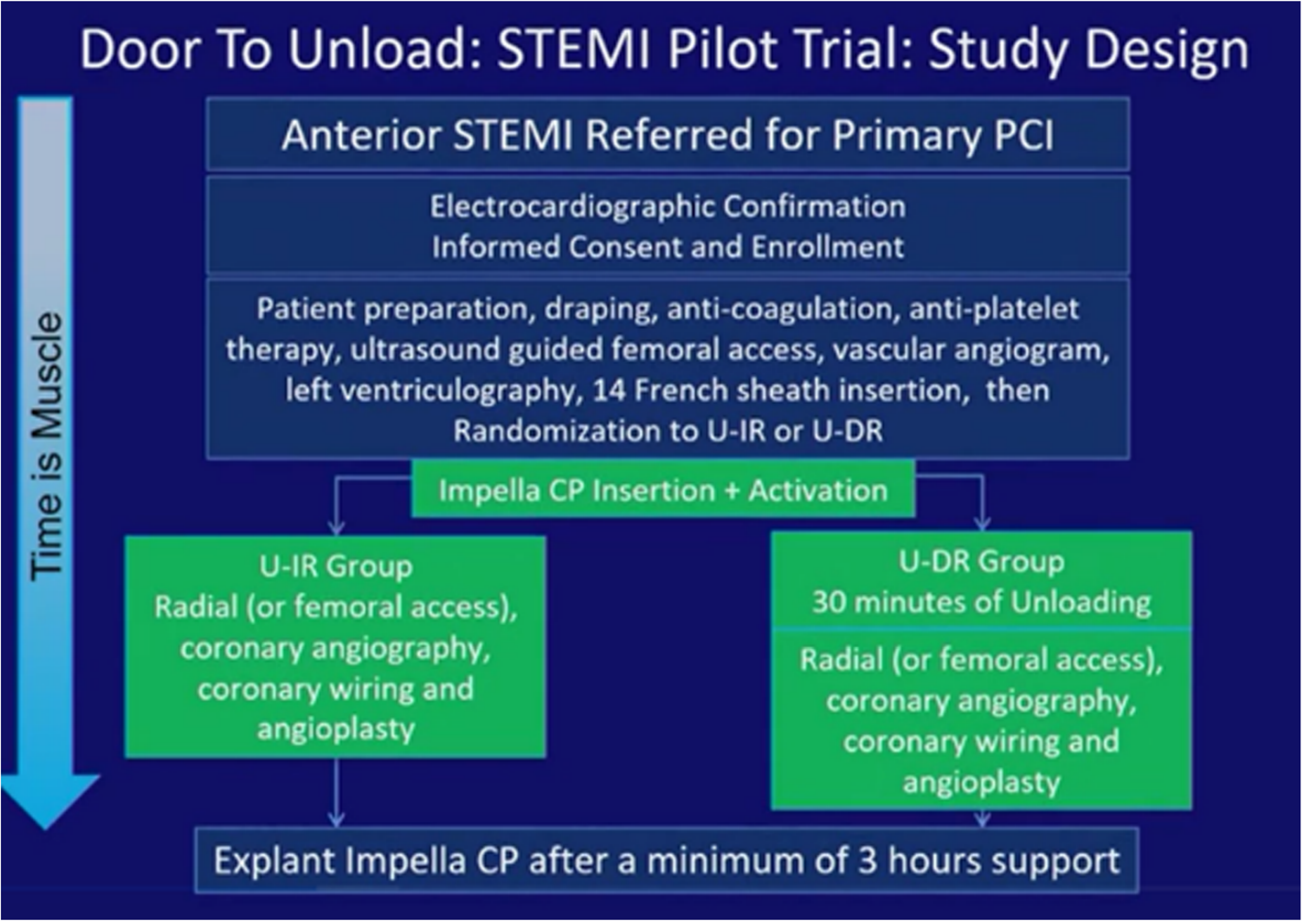
Study Design of the Door-To-Unload in STEMI Pilot Trial. U-DR = delayed reperfusion after 30 min of LV unloading by the Impella CP; U-IR = immediate reperfusion after placement of the Impella CP; CP = cardiac power

In this phase 1, multi-center, safety and feasibility pilot trial, 50 patients with anterior STEMI were randomized (1:1) to LV unloading using the Impella CP followed by immediate reperfusion (U-IR) or delayed reperfusion after 30 min of unloading (U-DR) (Fig. 9). Impella CP was explanted after a minimum of 3 h of support. The technical feasibility of the DTU in STEMI pilot trial was highly dependent on optimal team dynamics. Dr. Kapur stated that the pilot trial was successfully completed and the results will be presented at the late breaking science session at American Heart Association (AHA) Annual Meeting 2018 with a co-publication in Circulation [57].

Dr. Kapur highlighted that the DTU STEMI Pilot trial is the first step towards developing a physiologically integrated approach to acute AMI therapy. He further stated that the success of the DTU STEMI pilot and pivotal trials may pave way for testing additional investigations in conjunction with primary unloading, targeting both the ischemic as well as reperfusion injury to further reduce infarct size such as intravenous beta-blockade [58] and intracoronary vasodilators.

## Case report from the Door-to-Unload (DTU) in STEMI trial

### Presented by Haroon Faraz, MD

Dr. Faraz presented a clinical case from the Door-To-Unload (DTU) in STEMI pilot trial. In this phase 1, multi-center, safety and feasibility pilot trial, 50 patients with anterior STEMI were randomized (1:1) to LV unloading using the Impella CP followed by immediate reperfusion (U-IR) versus delayed reperfusion after 30 min of unloading (U-DR). Impella CP was explanted after a minimum of 3 h of support.

The patient was a 62-year-old morbidly obese male with a history of smoking who presented with chest pains that started about an hour prior to presentation. The patient had experienced a vague epigastric discomfort two days prior that spontaneously resolved, but recurred with greater intensity and was associated with shortness of breath and diaphoresis. Baseline LV ejection fraction (LVEF) was 43%. This patient was enrolled in the U-DR arm of the trial. Following successful LV unloading and percutaneous coronary intervention, the patient was discharged home on day 3. At 30 day follow-up, the patient’s LVEF had improved to 55%. Dr. Faraz noted that immediately after initiating LV unloading with the Impella CP ST-elevation began to resolve and the intensity of chest pain experienced by the patient had reduced, which reassured him of the benefits of LV unloading.

Dr. Faraz highlighted the challenges with enrolling and obtaining informed consent from patients experiencing anterior wall myocardial infarction (MI) for participation in the first exploratory study in humans testing the safety and feasibility of left ventricular (LV) unloading and delayed reperfusion as a method to reduce infarct size. He emphasized that choice of words regarding the purpose of the trial and positive reinforcement from the emergency department staff regarding the interventional cardiologist’s experience was vital in enrolling patients in the trial. He suggested a strategy of active 24/7 enrollment for the future pivotal trial and a dedicated champion at each clinical trial site to ensure coordination of multiple systems and processes needed for the successful completion of the trial.
